# Modulatory Activity of Uncaria tomentosa Extract in the Expression of Proteins Involved in the Unfolded Protein Response and Insulin Resistance

**DOI:** 10.3390/cimb48060624

**Published:** 2026-06-16

**Authors:** Bruna Freitas Marchi, Vittoria de Lima Camandona, Athirson Moraes Chanavat, Gustavo Roncoli Reigado, Carla Roberta de Oliveira Carvalho, Felipe Santiago Chambergo, Viviane Abreu Nunes

**Affiliations:** 1 Department of Chemistry and Molecular Biology, University of Gothenburg (GU), 413 90 Göteborg, Sweden; 2 Laboratory of Skin Physiology and Tissue Bioengineering, School of Arts, Sciences and Humanities, University of Sao Paulo (EACH-USP), São Paulo 03828-000, Brazil; 3 Laboratory of Aging and Molecular Biology, School of Arts, Sciences and Humanities, University of Sao Paulo (EACH-USP), São Paulo 03828-000, Brazil; 4 Department of Physiology and Biophysics, Institute of Biomedical Sciences, University of São Paulo (ICB-USP), São Paulo 05508-000, Brazil

**Keywords:** *Uncaria tomentosa*, palmitate, unfolded protein response, insulin resistance, type 2 diabetes mellitus

## Abstract

Type 2 diabetes mellitus (T2D) is associated with dyslipidemia, characterized by elevated plasmatic triglycerides and free fatty acids, particularly palmitate (PA), which may cause lipotoxicity in skeletal muscle cells. This leads to inflammation, activation of the unfolded protein response (UPR), insulin resistance, and cell death. Herbal medicines such as *Uncaria tomentosa* (UT) have shown potential as complementary treatments for T2D due to their protective effects. Purpose and study design: This study investigates the effect of UT aqueous extract on UPR and insulin resistance induced by PA in C2C12 myotubes. C2C12 myoblasts were grown in DMEM medium supplemented with 10% fetal bovine serum and differentiated into myotubes with 3.5% horse serum. The myotubes were incubated with 100 or 500 μM PA, 2–100 µM thapsigargin (Tg) or tunicamycin (Tn), in the presence or absence of 250 μg/mL UT extract or 100 µM TUDCA, for 2 or 6 h. The myotubes treated with UT extract for 6 h, after the incubation with 20 µM Tg, Tn or 500 µM PA, presented reduction in the expression of UPR-related genes ATF4 and CHOP by approximately 1.5-fold, and increased by 3-fold the expression of IRS-1, an insulin-signaling protein, when compared to myotubes incubated with only 20 µM Tg, Tn or 500 µM PA. These findings suggest that UT extract may serve as a modulator against skeletal muscle dyslipidemia by downregulating ATF4 and CHOP, reducing cell stress and death, while enhancing IRS-1 expression, which supports the use of the UT extract in managing insulin resistance and T2D.

## 1. Introduction

Type 2 diabetes mellitus (T2D) is a chronic disease that can lead to deterioration in the quality of life of individuals affected by it, due to the onset of various cardio- and microvascular complications, including poor wound healing, retinopathy, nephropathy, cognitive dysfunction, and periodontitis [1,2,3]. According to some authors [4], the global mortality rate caused by T2D and its complications in 2021 was 6.7 million.

In addition to being a syndrome of multiple etiologies, T2D can be characterized by chronic hyperglycemia with disturbances in the metabolism of carbohydrates, lipids, and proteins [5]. These disturbances result from changes in the production, secretion, and/or mechanism of action of insulin, insulin being the primary hypoglycemic hormone, whose primary target tissues are the liver, adipose tissue, and muscle, which may characterize the condition of insulin resistance or pre-diabetes, often associated with hypertension and dyslipidemia [2,5,6].

Diabetic dyslipidemia mainly consists of elevated plasma concentrations of triglycerides [7], and after the lipolysis process, free fatty acids are transported in the blood, mainly reversibly bound to albumin, being used primarily by skeletal and cardiac muscle tissue as an energy source [8,9].

The free fatty acid PA can be found in large quantities in diets derived from bovine products, such as meat and milk [10]. Furthermore, PA together with oleate constitute approximately 70–80% of total plasmatic free fatty acid [11,12]. The accumulation of PA in skeletal muscle leads to lipotoxicity, which may be associated with cell death or mitochondrial dysfunction, endoplasmic reticulum (ER) stress, and insulin resistance in insulin-dependent tissues such as skeletal muscle, liver, and adipose tissue [13,14].

In a condition where oxidative stress is not compensated, due to a hyperlipidic diet, for example, cells in the endoplasmic reticulum (ER) begin the formation of misfolded proteins, leading to the onset of ER stress [13,15]. If the formation of misfolded proteins persists, the condition of ER stress may culminate in the unfolded protein response (UPR), in which the protein eukaryotic initiation factor 2 alpha (eIF2α) reduces translational activity and overloading misfolded protein synthesis in the ER [15].

The phosphorylation of eIF2α mediated by PERK also promotes the selective induction of ATF4 [16,17], a protein that induces the expression of antioxidant genes that neutralize the increase in ROS produced during ER stress [18,19,20].

On the other hand, the PERK-ATF4 pathway can also drive pro-apoptotic responses by upregulating the C/EBP homologous protein (CHOP), a transcription factor linked to ER stress-induced apoptosis [17,21,22]. The CHOP is present in minimal concentrations in the cytosol of cells under redox state equilibrium, however, under conditions of ER stress and UPR activation, CHOP expression increases, and it accumulates in the cell nucleus [17,22].

As part of the UPR, the ER also signals molecular events involved in the expression of pro-inflammatory cytokines, such as tumor necrosis factor-alpha (TNF-α) [23]. This cytokine, for example, leads to the expression of c-Jun N-terminal kinase (JNK), which induces the phosphorylation of the serine residue on insulin receptor 1 (IRS-1) and inhibits its activity [24,25]. This results in decreased signaling through the IRS-1 receptors, impaired insulin binding, and the PI3K-AKT pathway, leading to an imbalance in metabolic activity and excessive cell loss, worsening the condition of insulin resistance in chronic diseases such as T2D [13,22,25].

In general, the use of combined therapies with drugs affecting lipid metabolism has been suggested for the treatment of individuals with various diseases, such as obesity and T2D.

In 2016, the National Center for Complementary and Integrative Health published a strategic plan to explore the science of complementary and integrative health, including phytotherapy [26]. Indeed, several plants such as *Uncaria tomentosa* (UT), known as an herbal medicine, have been used in therapies for the treatment of different diseases, such as arthritis, gastrointestinal diseases, viral infections and certain types of cancer [27,28]. In addition, UT has been associated with antioxidant, immunomodulatory and anti-inflammatory properties, which have increased scientific interest in its pharmacological potential [28,29,30,31].

The biological relevance of UT in metabolic dysfunction may be associated with its rich phytochemical composition, particularly oxindole alkaloids such as Mitraphylline, Isomitraphylline and Uncarine, as demonstrated in the chromatogram of the plant (Figure S1). These compounds exhibit anti-inflammatory activities that may directly modulate pathways involved in fatty acid-induced lipotoxicity and insulin resistance [31,32].

Oxindole alkaloid Mitraphylline and Uncarine derivatives have demonstrated immunomodulatory and anti-inflammatory effects, including inhibition of NF-κB, resulting in reduction in pro-inflammatory cytokines, mechanisms strongly associated with the attenuation of insulin resistance induced by saturated fatty acids [29,31,33].

Although UT has been extensively investigated for its antioxidant, anti-inflammatory, and immunomodulatory properties, few studies have specifically evaluated its modulatory effects on endoplasmic reticulum stress pathways in differentiated skeletal muscle cells exposed to palmitate-induced lipotoxicity. In particular, limited information is available regarding the regulation of ATF4, CHOP and IRS-1 expression under these metabolic stress conditions. Therefore, investigating the effects of the aqueous extract in this experimental model may contribute to a better understanding of the potential role of plant-derived bioactive compounds, in the modulation of insulin resistance-associated cellular stress.

According to some authors [29], it was shown in skeletal muscle cell cultures that the UT aqueous extract presents an antioxidant effect against palmitate-induced oxidative stress and cell death. Although evidence regarding the role of PA in inducing oxidative stress and insulin resistance is well reported, few studies have evaluated the modulatory potential of plant-derived bioactives on ER stress, particularly the ATF4-CHOP pathway of the UPR on differentiated myotubes.

Therefore, the aim of this study is to evaluate, in an in vitro model, the effects of UT aqueous extract on the activation of the UPR pathway, induced by PA in skeletal muscle cells, identifying ATF4/CHOP and IRS-1 proteins as key targets.

## 2. Material and Methods

### 2.1. Myoblasts Culture and Differentiation

The C2C12 skeletal muscle cell line (*Mus musculus*), sourced from the ATCC (American Type Culture Collection) and acquired from the Rio de Janeiro Cell Bank, was used. The cells were cultured in DMEM with a low glucose concentration, supplemented with 10% fetal bovine serum (proliferation medium) with the antibiotics penicillin/streptomycin at 10,000 IU/mL and 10 mg/mL, respectively, at 37 °C in a humidified atmosphere with 5% CO_2_, until the culture reached 80% confluence. For the differentiation of myoblasts into myotubes, using cell passage numbers from 9 to 14, the cultures were washed with phosphate-buffered saline (PBS), and the culture medium was replaced by DMEM supplemented with 3.5% horse serum (differentiation medium) for 21 days. The 3.5% horse serum concentration was employed based on prior optimization experiments evaluating concentrations ranging from 2% to 4%. Differentiation was conducted over 21 days, as low-glucose culture conditions are associated with slower myogenic progression. Morphological changes in the differentiated cultures were monitored daily using optical microscopy with a trinocular inverted microscope Nikon Eclipse TS100F (Nikon Instruments Inc., Melville, NY, EUA).

### 2.2. Analysis of Cell Differentiation by Fluorescence Microscopy

The expression of myogenic markers or staining with fluorescent dyes was analyzed after the differentiation period of 21 days in the C2C12 cultures. To block nonspecific binding sites, the samples were incubated with PBS containing 10% FBS for 20 min. Subsequently, the primary anti-desmin antibody (1:200) (Thermo Fisher, Waltham, MA, USA) was added and incubated for 12 h at 4 °C, protected from light. For visualization of positively stained cells, the secondary anti-IgG mouse antibody (IgG anti-mouse alexa fluor) (1:200), conjugated with green fluorescence FITC (GeneTex, Irvine, CA, USA), was used. The cells were washed and analyzed using a Zeiss AxioObserver D1 inverted fluorescence microscope (Carl Zeiss Microscopy GmbH, Oberkochen, Germany), after excitation at 480 nm and emission at 520 nm, using appropriate filters. Blue fluorescence was obtained from the nuclear marker dye Hoechst 33342 (Thermo Fisher, Waltham, MA, USA), after excitation at 350 nm and emission at 460 nm, while red fluorescence was obtained from the F-actin filament marker, ActinRed 555 (Thermo Fisher, Waltham, MA, USA), after excitation at 570 nm and emission at 590 nm. Both Hoechst33342 and ActinRed 555 were added simultaneously to the cells. Images were captured using the AxioVision 4.8 software (Carl Zeiss Microscopy GmbH, Oberkochen, Germany), with a magnification of 100×, an exposure time of 5400 ms, and analyzed with ImageJ software, version 1.53, evaluating the average fluorescence intensity of the images.

Finally, to confirm the differentiation of myoblasts into myotubes, the samples were subjected to analysis by real-time polymerase chain reaction (PCR), as described in Item 2.8.3, for the quantification of mRNA encoding proteins expressed during myogenesis. To optimize the presentation of the methods, the PCR section is only described in conjunction with the analysis of the expression of the genes of interest involved in the UPR.

### 2.3. Preparation of the Aqueous Extract of Uncaria tomentosa and Palmitate Fatty Acid Solutions

The powder of UT crude extract (Product batch number 100001372, code 2470), from the plant root bark, was donated by Herbarium Botanic Laboratory (herbarium.com.br, Curitiba, PR, Brazil). The chromatogram of the crude extract provided by the supplier (present in the Supplementary Material) [29], confirms the presence of some compounds, such as mitraphylline or isomitraphylline, typically used as confirmatory profiles to the species *tomentosa*. The crude extract compounds were purified by high-performance liquid chromatography (HPLC) for thirty-five minutes.

Aqueous extract preparation was selected based on the traditional medicinal use of UT as infusion/decoction. In addition, the phytochemical characterization of the extract (Figure S1) demonstrated the presence of bioactive oxindole alkaloids, including mitraphylline and isomitraphylline, confirmed by chromatographic analysis [29]. The same extraction and preparation protocol was consistently used throughout all experiments to improve reproducibility and standardization. The selection of the aqueous extract was also supported by previous findings demonstrating antioxidant and anti-lipotoxic effects in skeletal muscle cells [29].

To prepare the aqueous extract, UT crude plant extract was used at a concentration of 100 mg/mL, following the protocol described in [29]. The solution was prepared weekly using 100 mg of the extract in 1 mL of distilled water (dH_2_O), which was kept at 37 °C for 24 h. After this period, the solution was homogenized and centrifuged for 5 min, at 400× *g*. The supernatant was recovered and filtered (0.22 μm) to be used in the experiments.

The PA stock solution was prepared at a concentration of 0.1 M in absolute ethanol and was then diluted for final use in either the proliferation or differentiation medium, with 2% bovine serum albumin (BSA) added to conjugate the fatty acid to the protein.

### 2.4. Treatment of the Cells

The C2C12 cells were plated at a concentration of 2 × 10^5^ or 1 × 10^4^ cells/mL in 6-well or 96-well plates, respectively. After differentiation into myotubes, the cells were incubated for 24 h with the PA solution at different concentrations (100 and 500 μM), or with Tg ot Tn at different concentrations (5 and 20 µM).

These concentrations were selected based on previous studies [29,34]. In addition, previous reports have shown that concentrations between 2 and 20 µM of stressors, such as Tg and Tn, can induce cytotoxic effects [35,36]. Therefore, concentrations of 5 and 20 µM were chosen as ER stress-inducing conditions in differentiated myotubes.

After incubation, the samples were washed with PBS, followed by the addition of the aqueous UT extract, at a concentration of 250 μg/mL, for 2 or 6 h (therapeutic condition model) as described in [29] or followed by the addition of 100 µM TUDCA, for 6 h.

The control samples were used as the myotubes with no treatment in the culture medium. Control cultures received the corresponding vehicle solutions used in the experiments, including 0.05% ethanol as the final concentration present in the palmitate working solutions and DMSO for Tg-treated groups, at concentrations equivalent to those used in the treated samples.

### 2.5. Cell Viability Analysis by MTT

The colorimetric assay using 3-(4,5-dimethylthiazol-2yl)-2,5-diphenyltetrazolium bromide (MTT), which is metabolized only by viable cells, producing the blue formazan compound was used. The stock solution of MTT was prepared at a concentration of 5 mg/mL in PBS. After incubating the myotubes with PA or UT aqueous extract for 24 h, 5 µL of the MTT solution was added to the cultures in a 96-well microplate, and the cells were incubated for 4 h at 37 °C. After this period, the medium was removed, and 100 µL of dimethyl sulfoxide (DMSO) was added to each well to dissolve the formazan crystals. Cell viability was determined by reading the absorbance at 595 nm using the BioTek Synergy HT microplate reader (BioTek Instruments, Winooski, VT, USA). Four individual experiments were performed in triplicates and the results were analyzed using the Gen5 software, version 3.11 (https://www.agilent.com). For the construction of the graphs, absorbance was converted to percentage according to the presented formula, as per [29], considering the number of viable cells in each sample relative to the control.

$$Total\;\%\;=\left(\left(\frac{Abs\;sample}{Abs\;control}\right)\times 100\right)-100\%$$

The cytotoxicity was determined according to the classification by [37], as shown in Table 1.

### 2.6. Quantification of Reactive Oxygen Species

The amount of reactive oxygen species (ROS) in the myotubes was detected by fluorescence spectroscopy, using the fluorescent probe 2′,7′-dichlorofluorescein diacetate (DCFDA) from the commercial kit DCFDAd6883 (Sigma-Aldrich, St. Louis, MO, USA). After culturing the cells in 96-well plates, the medium was removed, and two washes with PBS were performed. Then, the DCFDA probe was added at 10 μM, diluted in DMEM without phenol red, and incubated for 30 min at 37 °C. Following this, the cultures were washed twice with PBS, and the presence of ROS was evaluated at λ_excitation_ = 485 nm and λ_emission_ = 528 nm, for 1 h kinetics in the BioTek Synergy HT microplate reader (BioTek Instruments, Winooski, VT, USA). The fluorescence results monitored between 20 and 50 min post-incubation with DCFDA were used, as the fluorescence peaks of standard samples with peroxide buffer were identified during the kinetic reaction (Figure S2). The results were expressed in arbitrary fluorescence units (AFU).

Three individual experiments were conducted in quadruplicates, and the results were analyzed using the Gen5 software, version 3.11 (https://www.agilent.com). The fluorescence values were expressed in arbitrary fluorescence units (AFU) and converted into percentages, considering the number of viable cells and the fluorescence of untreated cells in each sample relative to the control.

### 2.7. UPR Induction and Inhibition Solutions

In order to induce the UPR (unfolded protein response) and responses associated with ER stress in the myotubes, thapsigargin (Tg) was used as a positive control. Tg is a compound capable of inhibiting the activity of the sarco/endoplasmic reticulum calcium ATPase (SERCA ATPase), thereby promoting a reduction in calcium (Ca^2+^) ions in the ER lumen [38,39]. Additionally, tunicamycin (Tn) was used, which interferes with the inhibition of glycosylation at the amino (N-terminal) of proteins, leading to misfolding [40,41].

For control of UPR induction, stock solutions 1 mM Tg in dimethyl sulfoxide (DMSO) and 0.6 mM Tn in 95% ethanol (Sigma-Aldrich, St. Louis, MO, USA) were prepared. The cultures were incubated with Tg or Tn at different concentrations (5 and 20 μM), for 24 h, in either proliferation or differentiation culture medium, followed by two washes with PBS and incubation with 100 µM tauroursodeoxycholic acid (TUDCA), for 6 h, as a control for UPR inhibition, which was prepared as a stock solution at a concentration of 2 mM in distilled water (H_2_Od).

For the evaluation of cell viability, the colorimetric assay using MTT, as presented in Item 2.5, was employed. Four individual experiments were conducted in triplicates, and the results were analyzed using Gen5 software, version 3.11 (https://www.agilent.com). For graph construction, absorbance was converted into percentages according to the formula presented in Item 2.5, regarding the number of viable cells in each sample relative to the control.

### 2.8. Quantification of Gene Expression

#### 2.8.1. RNA Extraction

The RNA was extracted from the myoblasts and myotubes using the Trizol™ method (Invitrogen, Carlsbad, CA, USA), following the manufacturer’s instructions. After the different treatments, the cells were removed from the plates using 0.25% trypsin. Cell suspension was centrifuged for 5 min at 400× *g*. For RNA extraction, the cell pellet was resuspended in 500 μL of Trizol™, homogenized using a vortex, and frozen at −80 °C overnight. After this period, the samples were thawed at 30 °C for 5 min in a dry block. Subsequently, 250 µL of chloroform was added, and the samples were centrifuged at 12.000× *g* at 4 °C for 15 min. At the end, the RNA suspension (aqueous phase) was collected and transferred to a sterile microtube. Then, 500 µL of isopropyl alcohol was added, and the samples were incubated at 30 °C for 10 min. Centrifugation was performed at 12.000× *g* at 4 °C for 10 min, and after this period, the supernatant was discarded. The RNA pellet was resuspended in 600 µL of 70% ethanol and centrifuged at 7.500× *g* at 4 °C for 15 min. At the end, the supernatant was discarded, and the RNA samples were resuspended in 40 µL of ultrapure water. The RNA concentration was measured in ng·µL^−1^ by absorbance at wavelengths of 260 and 280 nm using a Nanodrop^®^ spectrophotometer (Thermo Fisher, Waltham, MA, USA).

#### 2.8.2. Reverse Transcription to Produce the cDNA Strip

The first cDNA strand was synthesized using the SuperScript^®^ III First-Strand Synthesis System for RT-PCR kit (Invitrogen, Carlsbad, CA, USA), following the manufacturer’s instructions. After quantification, 2 µg of RNA was used to synthesize the cDNA strand in two steps: the first step involved the removal of genomic DNA by incubating 8 μL of RNA (2 μg), 1 μL of oligo(dT) 20 primer, and 1 µL of the 10 mM deoxyribonucleotide triphosphates (dNTPs) mix at 65 °C for 5 min, the second step referred to reverse transcription, in which 10 μL of the product from the first step, 2 μL of 10× reaction buffer, 4 μL of 25 mM magnesium chloride (MgCl_2_), 2 µL of 0.1 M dithiothreitol (DTT), 1 µL of RNase (40 U/µL), and 1 µL of SuperScript III reverse transcriptase (200 U/µL) were added, totaling 20 μL. This solution was incubated at 65 °C for 5 min in a Veriti^®^ thermal cycler (Life Technologies™, Carlsbad, CA, USA) as per the manufacturer’s recommendations. Subsequently, the samples underwent one cycle of 4 °C for 5 min, followed by 50 °C for 50 min, and 85 °C for 5 min, finalizing with stabilization at 4 °C for 10 min.

#### 2.8.3. Evaluation of the Expression of Genes by Real-Time PCR

To quantify the mRNA of the genes of interest, the fluorescent reagent PowerUp™ SYBR^®^ Green Master Mix (Thermo Fisher, Waltham, MA, USA) was used. For each sample, a solution was prepared containing 5 µL of PowerUp™ 0.4 µL of the forward primer and 0.4 µL of the reverse (100 µM), 3.2 µL of ultrapure MilliQ H_2_O, and 1 µL of the cDNA of interest. The temperature for the first step of DNA denaturation was set to 95 °C for 10 min. Then, the samples underwent 40 cycles of denaturation at 95 °C for 30 s, annealing at 60 °C for 1 min, and extension at 72 °C for 1 min. The reactions were developed in the Eco™ Real-Time PCR System (Illumina, San Diego, CA, USA) and the data obtained were analyzed using Eco™ software, version 4.1^®^. The expression of the genes ATF4, CHOP and IRS-1 was normalized regarding the expression of the housekeeping gene glyceraldehyde 3-phosphate dehydrogenase (GAPDH), used as internal control for cDNA normalization. For quantification, the expression levels were calculated by using the 2^−ΔΔCt^ method.

The oligonucleotides, shown in Table 2, were designed to avoid non-specific annealing and in compliance with recommendations for use in PCR.

### 2.9. Analysis of Protein Expression by Western Blotting

#### 2.9.1. Cell Extracts Collection

After the respective treatments, the myotubes were removed from the plates with 0.25% trypsin. The plates were then washed with PBS, and the cell suspension was centrifuged for 5 min at 400× *g*. For protein extraction, the supernatant was discarded. The cell pellet was lysed with lysis buffer (HEPES 25 mM, pH 7.4, EDTA 2 mM, Triton X-100 0.5%) in the presence of a cocktail of protease and phosphatase inhibitors, containing 4-(2-aminoethyl)-benzenesulfonyl fluoride (AEBSF) hydrochloride, aprotinin, bestatin, E-64, leupeptin, and pepstatin A, 100× (Sigma-Aldrich Corporation, St. Louis, MO, USA). The cell suspension was frozen at −80 °C, thawed, and homogenized. The cellular extracts were then centrifuged at 12.000× *g*, 4 °C, for 20 min. The supernatant was collected and used in assays to assess the expression of proteins involved in transcription, translation, and apoptosis regulation pathways (ATF4 and CHOP), as well as in the insulin signaling pathway (IRS-1).

#### 2.9.2. Quantification of Proteins

To determine the protein content in each sample, the Bradford method [50] was used. Aliquots of 5 μL of the supernatant were diluted in 15 µL of ultrapure H_2_O and added to a 96-well microplate, along with 180 μL of Bradford reagent (Nova Biotecnologia, BR). The samples were then read at 595 nm using the BioTek Synergy HT microplate reader, and the protein concentration was determined by extrapolating the values from a standard curve of bovine serum albumin (BSA).

#### 2.9.3. Western Blotting (WB)

Aliquots containing 30 µg of protein were added to 10 µL of sample buffer (Tris 500 mM pH 6.8, glycerol 10%, SDS 10%, β-mercaptoethanol 10%, and bromophenol blue 0.05%) and separated by two different concentrations of polyacrylamide gel electrophoresis (PAGE), 8% for IRS-1 protein identification and 12% for ATF4 or CHOPs, in the presence of sodium dodecyl sulfate (SDS-PAGE) 10%, at 100 V for 1.5 h. After gel separation, the proteins were transferred to a nitrocellulose membrane, which was blocked for 1 h with a blocking solution (Tris 100 mM, pH 7.3, and 5% skimmed milk powder). After blocking, the membrane was incubated overnight with primary antibodies of interest (1:1000): anti-ATF4 (code 11815S), anti-CHOP (code 2895S), or anti-IRS-1 (code 2382S) (Cell Signaling Technology, Danvers, MA, USA), and subsequently incubated for 2 h at room temperature with secondary antibody anti-IgG from rabbit or mouse conjugated with peroxidase (1:1000). The proteins were identified using the Amersham ECL detection reagent (GE Healthcare, Mascot, NSW, Australia), under exposure to chemiluminescent light for 20 secs. Protein bands were captured using the C-DiGit Blot Scanner (LICORbio, Lincoln, NE, USA). Densitometric analysis was performed using GelAnalyzer 19.1 software (www.gelanalyzer.com) from the two independent experiments. For each sample, the target protein signals were divided by their corresponding β-actin signal and the protein quantification was normalized by the average ratio of the control sample to find fold changes. The results were obtained from at least two independent experiments.

### 2.10. Statistical Analysis

All results were expressed as the mean ± standard deviation and differences were assessed by two-way ANOVA, followed by the Bonferroni post-test, using GraphPad Prism software, version 8 (GraphPad Software Inc., San Diego, CA, USA, www.graphpad.com). Differences were first considered significant when *p* < 0.05, and after multiple comparisons, the differences were considered significant when *p* < 0.01.

## 3. Results

### 3.1. Proliferation of Myoblasts and Differentiation into Myotubes

**Figure 1:**
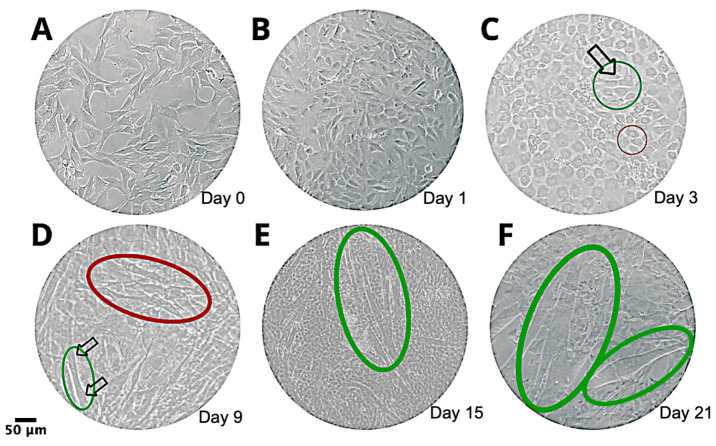
Induction of myoblast differentiation into myotubes. (**A**) Myoblasts cultured in proliferation medium; (**B**) 1st day of the initiation of differentiation induction; (**C**) 3rd day of differentiation. In the red highlight, fused myoblasts are shown, and in green, fused myotubes are shown; (**D**) 9th day of differentiation. In the green highlight, a myotube is shown, and in red, the alignment between myotubes is shown; (**E**) 15th day of differentiation. In the green highlight, more elongated myotubes are shown, and in greater quantities on the plate; (**F**) 21st day after the initiation of differentiation induction. In green, a mature differentiated myotube is demonstrated, according to [51], where the diameter of the myotubes is ≥50 µM. Magnification 100×, using a Nikon TS100F inverted microscope, scale: 50 µm.

The morphology of myoblasts on the first day of culture in proliferation medium (day 0) is demonstrated in Figure 1A. The myoblasts exhibited elongated and multinucleated morphology (Figure 1E,F), characteristic of mature myotubes, as described in [51].

Figure 1B displays the appearance of myoblasts on the first day in differentiation medium. By day 3 (Figure 1C), fused myoblasts can be observed in the red-highlighted region, while early-stage myotube formation is seen in the green-highlighted region, consistent with descriptions by [52]. By day 9 (Figure 1D), the differentiated myotubes begin to align (red region), and the fusion process becomes more evident (green region). At day 15 (Figure 1E), myotubes appear more elongated, and by day 21 (Figure 1F), mature myotubes with ≥50 µm diameters are observed, in accordance with some authors [51].

#### 3.1.1. Evaluation of Myogenic Differentiation into 2D Myotubes by Fluorescence Microscopy

**Figure 2:**
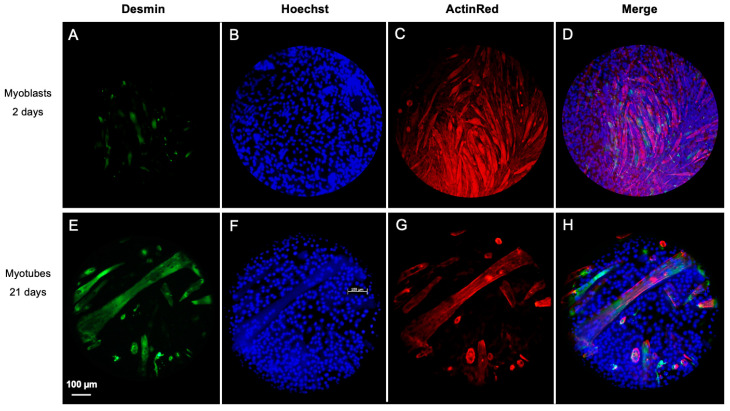
Expression of desmin in myoblasts and differentiated myotubes. (**A**,**E**) Desmin labeling (green); (**B**,**F**) Nucleus labeling with Hoechst 33342; (**C**,**G**) Cytoplasm labeling with ActinRed; (**D**,**H**) Merged pictures from each group of cells. Magnification 200×, using a Zeiss fluorescence microscope, scale bar: 100 µm.

After 21 days of differentiation, desmin expression was analyzed by immunofluorescence. In myoblast cultures, only few regions expressed desmin (Figure 2A), whereas myotubes showed a significantly higher expression (Figure 2E).

The fluorescent marker Hoechst 33342 (Figure 2B,F) was used for nuclear staining, while ActinRed (Figure 2C,G) labeled cytoplasmic actin filaments. The overlay of all three markers (Figure 2D,H) confirmed myotube formation. Images were captured using a Zeiss AxioObserver D1 fluorescence microscope at 100× magnification, with scale bars set at 100 μm.

#### 3.1.2. Evaluation of the Expression of Genes Involved in Myogenesis by PCR, in Myoblasts and Myotubes

To confirm the differentiation process, real-time PCR was performed to quantify the expression of PAX7, MyF5, MyoD1, Myog, and MyF6/MRF4, genes involved in myogenesis.

**Figure 3:**
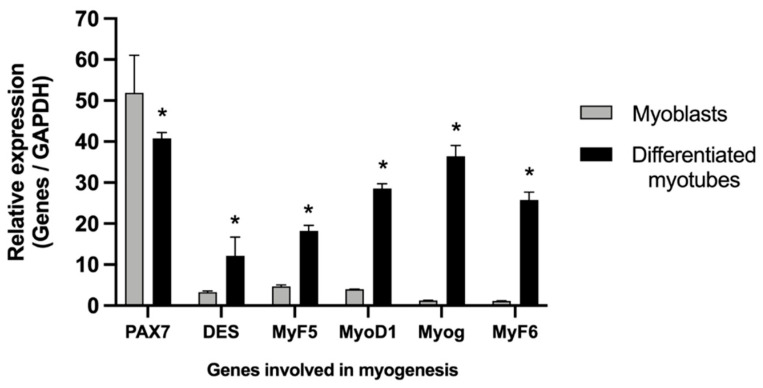
Relative expression of genes involved in myogenesis in two different cell culture models. Myoblasts were cultured for 2 days, and myotubes were cultured for 21 days. The relative expression of the genes was normalized to the expression of the constitutive gene GAPDH, with the myoblast sample considered as the control. The data are presented with values from two experiments in duplicates. The differences were analyzed by two-way ANOVA, followed by Bonferroni post-test. *, indicates a significant difference compared to the myoblasts (*p* < 0.05).

It was observed that the PAX7 gene was expressed in both myoblast and differentiated myotube cultures. However, there was a greater difference in expression in the myotubes, with a reduction of about 22% compared to the myoblasts (Figure 3).

The expression of the gene encoding the desmin protein (DES) was 4-fold higher in the myotube cultures compared to the myoblasts. Regarding the genes MyF5, MyoD1, and Myog, a higher expression was observed in the myotube cultures, with increases of four, seven, and 30 times, respectively, compared to the myoblasts.

Finally, the expression of the MyF6 gene, also known as MRF4, was evaluated, showing a 23-fold increase in the myotube cultures compared to the myoblasts (Figure 3).

### 3.2. Quantification of ROS Production in Differentiated Myotubes

Based on a prior study [29], the therapeutic effect of aqueous extract was tested in cultures after differentiation into myotubes.

**Figure 4:**
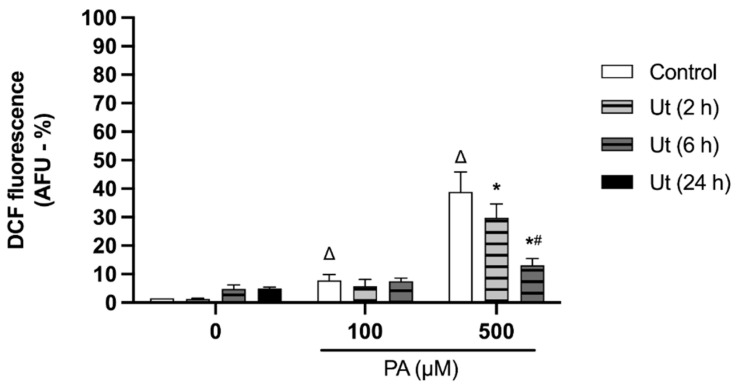
Quantification of ROS in differentiated myotubes incubated with palmitate (PA), in the absence or presence of UT aqueous extract. The myotubes were incubated with PA at different concentrations, followed by treatment with UT aqueous extract for 2 or 6 h. The samples were evaluated by fluorescence spectroscopy, at excitation wavelength λ = 485 nm and emission wavelength λ = 528 nm. ROS formation was measured in arbitrary fluorescence units (AFU) and expressed as a percentage, considering the number of viable cells in each sample, relative to the control (PA 0 µM). The data are presented as the mean ± SEM of three individual experiments in quadruplicates. Differences were analyzed by two-way ANOVA, followed by the Bonferroni post-test. ∆ indicates a difference compared to the control (PA 0 µM). *, indicates a difference compared to the control of each group; #, indicates a difference between the two incubation periods with UT aqueous extract for 2 or 6 h (*p* < 0.05).

Incubation of myotubes with 100 µM PA, regardless of the UT aqueous extract treatment for 24 h, did not affect ROS formation compared with control (Figure 4). However, when the cultures were incubated with 500 µM PA, ROS formation increased approximately 40-fold relative to the control. This reinforces the role of PA in ROS production and possibly oxidative stress.

Treatment of the myotubes with UT aqueous extract for 2 h resulted in 25% reduction in ROS production, compared to cultures exposed only to 500 µM PA. Furthermore, treatment with the extract for 6 h under the same therapeutic condition promoted 62.5% reduction in ROS formation, compared to the control (Figure 4).

As the results demonstrated the treatment with UT extract for 6 h had a better influence on cell viability recovery and decreased ROS formation by approximately 40%, in comparison to the treatments performed in 2 h, it was decided to keep the 6 h treatment approach with the UT extract, and use the same approach with TUDCA treatment in the following experiments.

### 3.3. UPR Induction and Inhibition

To assess ER stress responses, myotubes were incubated with Tg or Tn at 5 or 20 μM. In addition, 100 µM TUDCA was used as a UPR inhibitor.

**Figure 5:**
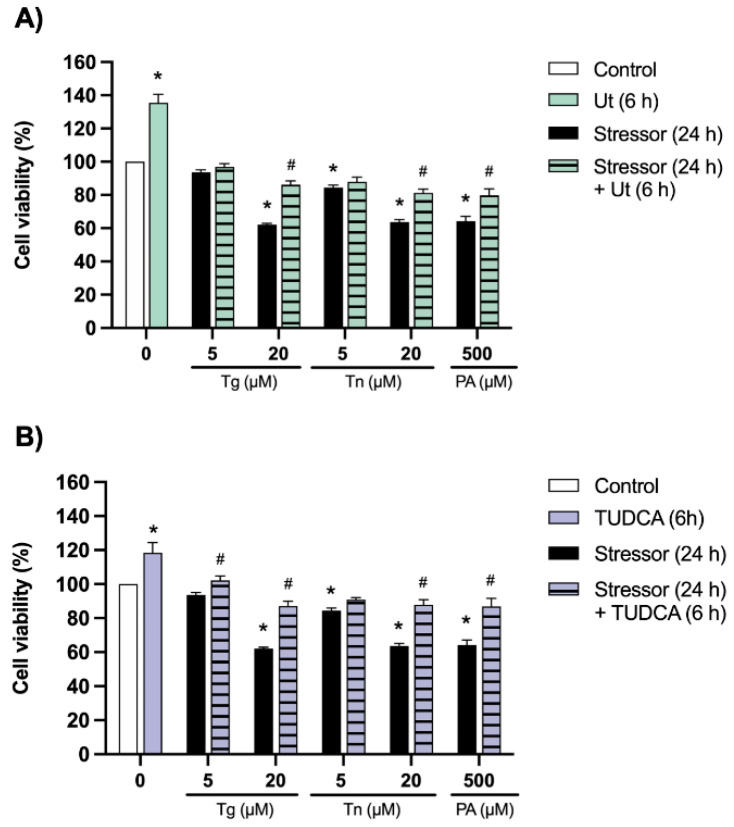
Effect of UT aqueous extract and TUDCA on the metabolic activity of myotubes induced by thapsigargin (Tg), tunicamycin (Tn), or palmitate (PA). (**A**) The cultures were incubated with solutions of Tg or Tn at concentrations of 5 or 20 µM, or PA 500 µM, for 24 h, followed by washing with PBS and then treated with the UT aqueous extract (250 μg/mL) for 6 h; (**B**) The cultures were incubated under the same conditions as above, washed with PBS, and treated for 6 h with TUDCA (100 µM). The cell cultures were evaluated by the MTT method. The values are expressed as a percentage relative to the control (0 µM), according to the number of viable cells in each sample. The data are presented as the mean ± SE of three individual experiments in quadruplicates. Differences were analyzed by two-way ANOVA, followed by the Bonferroni post-test. * indicates difference compared to the control (0 µM). # indicates difference compared to the 24 h stressor in each group (*p* < 0.05).

In cultures incubated for 24 h with 20 µM (Tg) and treated with UT, a 19% increase in metabolic activity was observed relative to the control (Figure 5A). Similarly, cultures incubated with 20 µM (Tn) and treated with UT showed a 26% increase in viability compared with cultures incubated only with the stressor. When cultures were incubated with 500 µM PA followed by a 6 h treatment with UT, a 17% increase in viability was observed compared to untreated cultures.

In addition, the protective effect of TUDCA on the metabolic activity of myotubes incubated with Tg or Tn was evaluated (Figure 5B). In cultures incubated with 5.0 µM or 20 µM Tg, followed by TUDCA treatment, 9% and 21% increases in viability, respectively, were observed compared to untreated cultures. In cultures incubated with 20 µM Tn and treated with TUDCA, a 37% increase in metabolic activity was observed. Treatment with TUDCA for 6 h in cultures incubated with 500 µM PA resulted in a 26% increase in viability compared to untreated cultures.

It is important to note that the MTT assay reflects mitochondrial metabolic activity rather than direct cell number or proliferation. Therefore, the reduction in MTT signal observed under PA, Tg or Tn treatment indicates impaired cellular metabolic activity associated with ER stress, rather than solely reduced cell proliferation.

**Figure 6:**
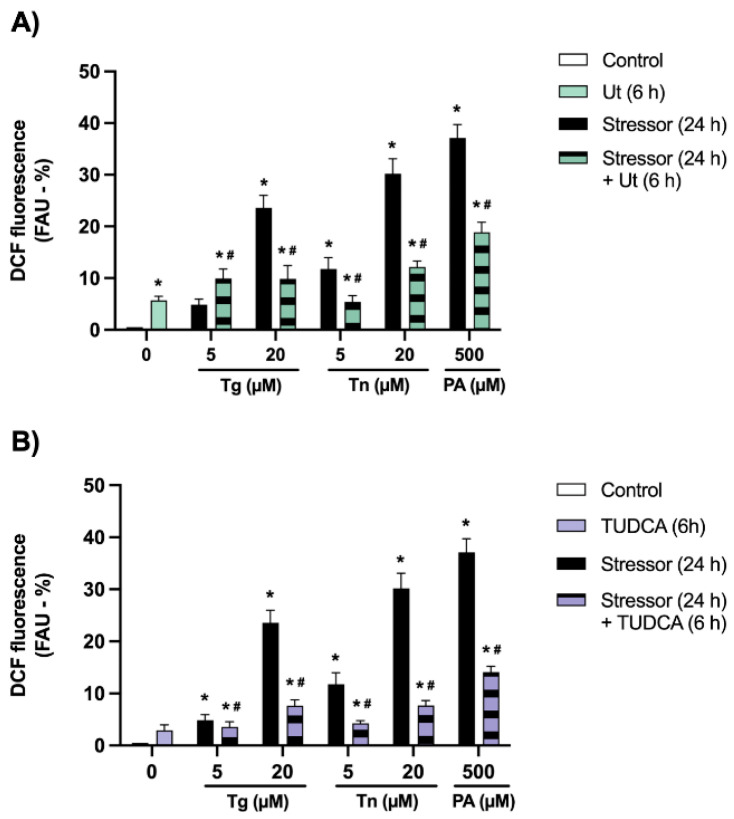
Quantification of ROS production in myotubes. The cultures were incubated with thapsigargin (Tg), tunicamycin (Tn), or palmitate (PA) for 24 h, with or without treatment with (**A**) UT 250 µg/mL; or (**B**) TUDCA 100 µM, for 6 h. The samples were evaluated by fluorescence spectroscopy, at excitation wavelength λ = 485 nm and emission wavelength λ = 528 nm. ROS formation was measured in fluorescence arbitrary units (FAU) and expressed as a percentage, based on the number of viable cells in each sample, relative to the control (0 µM). The data are presented as mean ± SE of three individual experiments in quadruplicates. Differences were analyzed by two-way ANOVA, followed by the Bonferroni post-test. * indicates difference compared to the control (0 µM). # indicates difference compared to the 24 h stressor in each group (*p* < 0.05).

When quantifying ROS production induced by the ER stressors, Tg or Tn, in the presence or absence of the UT aqueous extract, in myotube cultures, it was observed that incubation with 5.0 µM Tg, followed by treatment with UT aqueous extract for 6 h, promoted a 66% increase in ROS production compared to untreated cultures (Figure 6A). In cultures incubated with 20 µM Tg and treated with UT, a reduction of about 57% in ROS production was observed compared to the untreated control.

Regarding cultures incubated with 5.0 µM Tn followed by UT treatment, a reduction of approximately 55% in ROS production was observed, while cultures incubated with 20 µM Tn and treated with UT showed a 60% reduction in ROS production compared to cultures incubated only with the stressor. In cultures incubated with 500 µM PA and treated with UT for 6 h, there was a 59% reduction in ROS production compared to untreated cultures (Figure 6A).

Additionally, the effect of TUDCA on cultures incubated with Tg or Tn was evaluated for comparison (Figure 6B). In cultures incubated with 5.0 µM Tg and treated with TUDCA for 6 h, there was a 30% reduction in ROS production compared to untreated cultures. In cultures incubated with 20 µM Tg followed by TUDCA treatment, a reduction of approximately 63% in ROS production was observed compared to untreated cultures.

For cultures incubated with 5.0 µM or 20 µM Tn, followed by TUDCA treatment, reductions of approximately 55% and 74% in ROS production, respectively, were observed. In cultures incubated with 500 µM PA, the treatment with TUDCA for 6 h resulted in a 62% reduction in ROS production compared to untreated cultures (Figure 6B).

### 3.4. Quantification of UPR Gene Expression in Differentiated Myotubes

The PCR results show that PA, Tg and Tn increased ATF4 and CHOP expression, while IRS-1 expression decreased.

**Figure 7:**
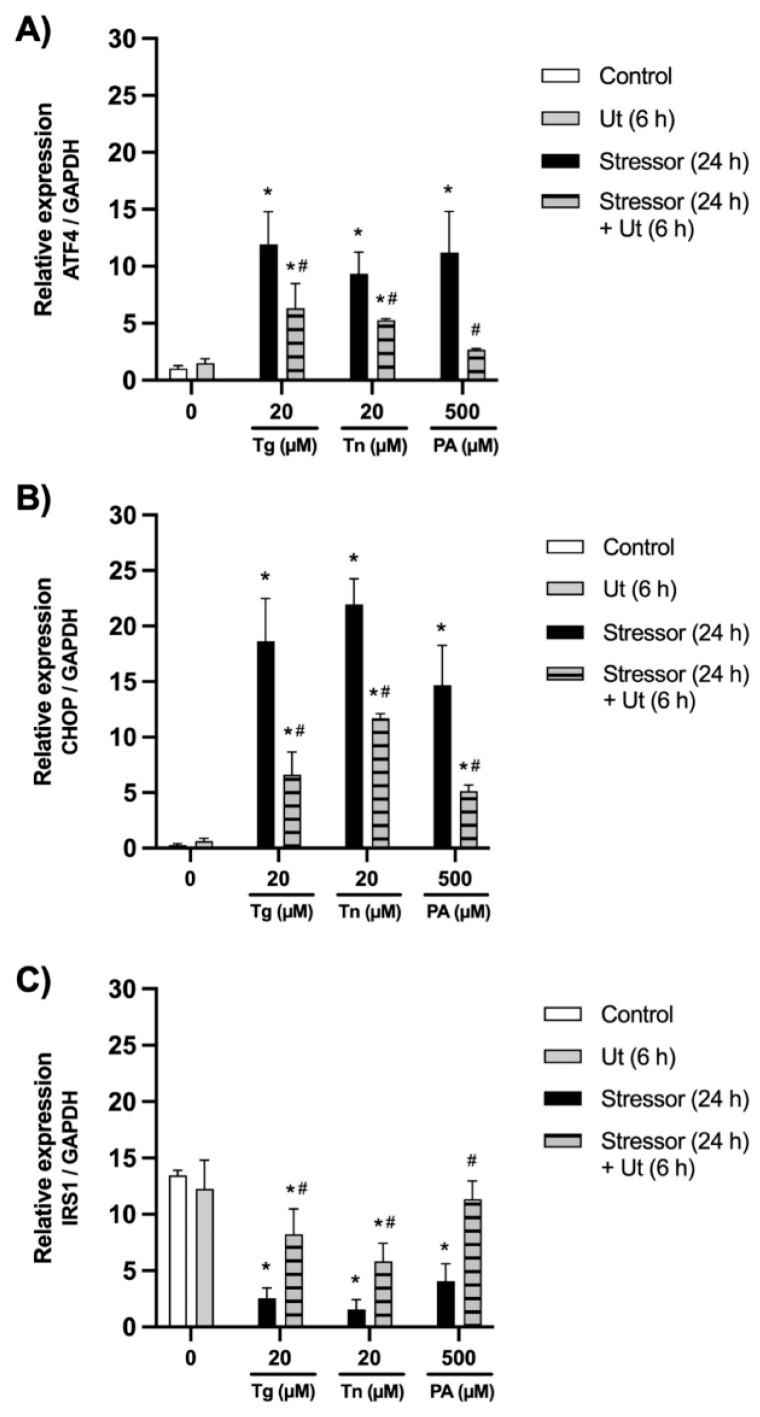
Relative expression of the genes. (**A**) ATF4; (**B**) CHOP; (**C**) IRS-1 in myotubes. The relative expression of the target genes was normalized according to the expression of the housekeeping gene GAPDH, with the untreated sample (0 µM) considered as the control. The data are presented as mean ± SE of two individual experiments in duplicates. Differences were analyzed by two-way ANOVA, followed by the Bonferroni post-test. *, indicates a difference compared to the control (0 µM). #, indicates a difference compared to the 24 h stressor of each group (*p* < 0.05).

Specifically, in the myotube cultures, ATF4 gene expression was 12 times higher in the cultures incubated with Tg 20 µM compared to the control. However, when these cultures were treated with the UT aqueous extract for 6 h, there was a 2-fold reduction in expression compared to the cultures incubated only with Tg 20 µM (Figure 7A). For cultures incubated with Tn 20 µM, the expression of the ATF4 gene was nine times higher compared to the control. Similarly, treatment with the UT reduced ATF4 expression by 1.8 times, compared to the cultures incubated only with Tn.

In cultures incubated solely with 500 µM PA, there was an 11-fold increase in ATF4 expression compared to the control. Treatment with UT aqueous extract after PA incubation resulted in a 4-fold reduction in the expression of this gene compared to incubation with only 500 µM PA.

Regarding the CHOP gene, it was expressed 18.5 times higher in cultures incubated with 20 µM Tg compared to the control. Interestingly, treatment of these cultures with the UT for 6 h led to a reduction of approximately 3-fold in CHOP expression compared to cultures incubated only with 20 µM Tg (Figure 7B). In cultures incubated with 20 µM Tn, the expression of the CHOP gene increased 21-fold compared to the control. When these cultures were treated with UT aqueous extract, there was a 1.7-fold reduction compared to cultures incubated with only Tn.

Comparatively, in cultures incubated only with 500 µM PA, there was a 14.7-fold increase in CHOP expression compared to the control, whereas treatment with UT aqueous extract after PA incubation resulted in a 2.9-fold reduction in CHOP expression compared to cultures incubated with only 500 µM PA.

Furthermore, the expression of the IRS-1 gene, involved in the insulin signaling pathway, was investigated. The IRS-1 gene was expressed 5.5 times less in cultures incubated with 20 µM Tg compared to the control. Treatment of the cultures with the UT for 6 h resulted in a 3-fold increase in expression compared to cultures incubated only with 20 µM Tg (Figure 7C). When the cells were incubated with 20 µM Tg, there was a 6.5-fold decrease in the expression of the IRS-1 gene compared to the control. Subsequent treatment with UT aqueous extract resulted in a 2.8-fold increase in the expression of this gene compared to cultures incubated only with 20 µM Tn.

Finally, in cultures incubated with 500 µM PA, there was a 3.5-fold reduction in IRS-1 expression compared to the control. However, IRS-1 expression was increased 3-fold after treatment with the UT following incubation with PA.

### 3.5. Determination of Protein Expression in Differentiated Myotubes by Western Blotting

**Figure 8:**
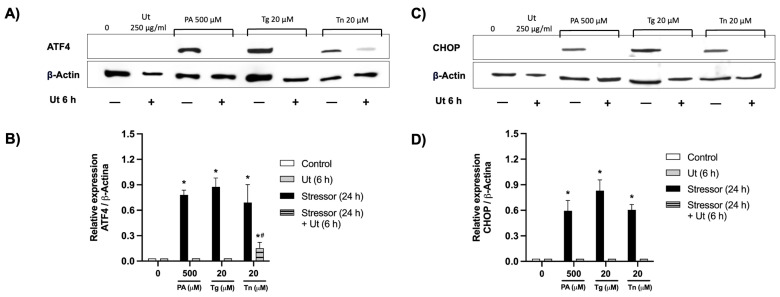
Expression of the ATF4 and CHOP proteins in myotubes differentiated for 21 days. (**A**) ATF4 expression relative to the control protein β-actin, by WB. (**B**) Densitometry of the relative expression of ATF4. (**C**) Expression of CHOP relative to the control protein β-actin, by WB. (**D**) Densitometry of the relative expression of CHOP. Data are presented as mean ± SE of two individual experiments. Differences were analyzed by two-way ANOVA, followed by Bonferroni post-test. *, indicates a difference compared to the control (0 µM). #, indicates a difference compared to the 24 h stressor of each group (*p* < 0.05).

The expression of ATF4 was evaluated (Figure 8A,B), and an increase in protein expression was observed, with a 16-fold rise in differentiated myotube cultures incubated with 500 µM PA (Figure 8B). Additionally, when comparing the amount of ATF4 in cultures incubated with Tg and Tn, higher expression was observed, approximately 18 and 14 times higher, respectively, compared to the control. Even after 6 h of treatment with UT aqueous extract, in myotube cultures incubated with Tn, ATF4 expression was still detected, but with a reduction of about 3.5 times in protein expression compared to the control treated only with the Tn stressor.

Thus, these results for ATF4 expression are similar to those obtained from the PCR assay in myotubes, where incubation of the samples with only 500 µM PA resulted in a 13-fold increase in protein expression, and the therapeutic treatment with UT reduced ATF4 expression by 4 times (Figure 7A).

The expression of the homologous protein C/EBP (CHOP) was also assessed (Figure 8C,D). An expression approximately 12 times higher for CHOP was observed in differentiated myotube cultures incubated with 500 µM PA (Figure 8D). However, when comparing the presence of CHOP in cultures incubated with the ER stressors, a difference was observed in the cultures incubated with Tg, which exhibited a 16-fold higher expression of the protein compared to the control. Similarly, to the incubation with PA, cultures incubated with Tn also showed an expression approximately 12 times higher for CHOP compared to the control.

As observed for ATF4, the results for CHOP expression are similar to those obtained in the PCR assay in myotubes, where incubation with only 500 µM PA increased protein expression around 15 times (Figure 7B).

**Figure 9:**
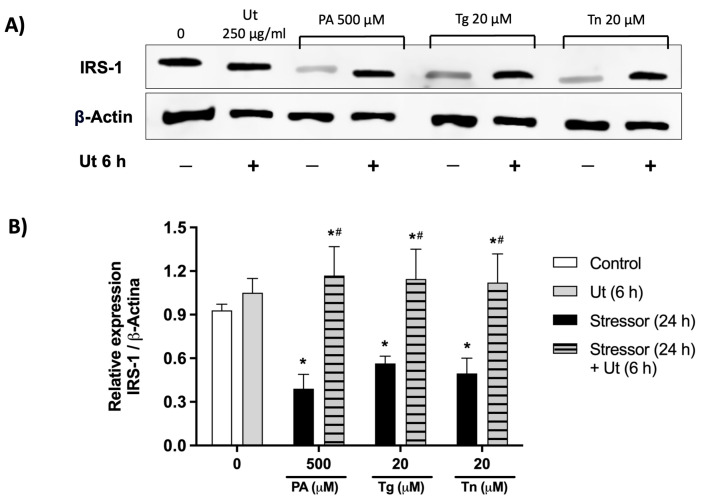
Quantification of IRS-1 protein in myotubes. (**A**) Relative expression of IRS-1 in relation to the control protein β-actin, by WB, in differentiated myotubes for 21 Days. (**B**) Densitometry of relative IRS-1 expression. The data are presented as mean ± standard error of the mean (SE) from two individual experiments. Differences were analyzed by two-way ANOVA, followed by the Bonferroni post-test. *, indicates a difference compared to the control (0 µM). #, indicates a difference compared to the stressor for 24 h in each group (*p* < 0.05).

Finally, the expression of IRS-1 was assessed by Western blotting (Figure 9A,B). In the myotube cultures incubated with PA, a 2.5-fold decrease in protein expression was observed compared to the control (Figure 9B). However, in cultures treated under the therapeutic condition with UT aqueous extract, a 3-fold increase in IRS-1 expression was observed.

Incubation of the cultures with Tg and Tn resulted in a 1.5 to 2-fold reduction in protein expression compared to the control. On the other hand, when these cultures were treated with UT aqueous extract, there was approximately a 2-fold increase in IRS-1 expression compared to the untreated samples (Figure 9B).

The results of IRS-1 expression by WB were also similar to those obtained in PCR, where incubation with only 500 µM PA reduced protein expression by 3 times, and the therapeutic treatment with UT increased IRS-1 expression by approximately 3 times (Figure 7C).

## 4. Discussion

This study demonstrates that UT aqueous extract significantly reduces oxidative and ER stress, while improving insulin signaling in skeletal muscle myotubes exposed to lipotoxic conditions. These findings are relevant given the central role of skeletal muscle in T2D pathogenesis and the lack of effective adjuvant therapies targeting cellular stress pathways [13,29,30].

Myoblasts, which act as satellite cells in early maturation, are crucial for evaluating muscle regeneration and repair in tissue damage models [53,54]. Differentiation was confirmed by increased expression of desmin and myogenic regulatory factors (e.g., MyoD, Myogenin), consistent with skeletal muscle maturation [55,56].

In myoblast cultures, only small regions of the cultures expressed the protein desmin, whereas in myotubes, desmin was significantly upregulated, aligning with the structural transformation associated with skeletal muscle development. Additionally, PCR analysis confirmed the expression of myogenic regulatory genes, including MyoD, Myf6/MRF4, Myf5, and Myogenin, which are essential for muscle fiber maturation and contractility [57,58]. The marked increase in Myf6/MRF4 and Myogenin expression in myotubes further supports their differentiation into functionally mature cells [54].

According to some authors [53,54], myoblasts differentiate into myotubes by expressing important myogenic regulatory factors such as MyoD, Myf6/MRF4, Myf5, and Myogenin, which aid in the formation of specialized fibers with contractile capacity. The results demonstrate that after 21 days of myotube differentiation, the genes MyoD, Myf6/MRF4, Myf5, and Myogenin, which are involved in myogenesis, were expressed at least 10 times more than in myoblasts without differentiation stimuli. The differentiation process with significant expression of the genes Myf6/MRF4 and Myogenin provides greater structural consistency to the myotubes [54,57,58].

In the next step, the cytotoxic effect of PA on myotubes was evaluated, both in the absence and presence of treatment with UT aqueous extract. Indeed, we observed that 500 μM PA significantly increased ROS levels, supporting its role in oxidative stress and metabolic dysfunction. On the other hand, according to [29], the treatment with UT aqueous extract at a concentration of 250 μg/mL promoted an approximately 45% increase in viability compared to the control. Thus, these concentrations of PA fatty acid and UT were used in this study.

Accordingly, the recovery of the MTT signal after UT treatment indicates preservation of cellular metabolic activity and viability, consistent with attenuation of ER stress and restoration of insulin signaling, rather than a proliferative effect.

Following 2 h or 6 h of UT aqueous extract treatment, ROS levels were reduced by around 25 and 62.5%, respectively, compared to untreated palmitate-exposed cells. This suggests that UT aqueous extract exerts a time-dependent effect while acting as an antioxidant agent, likely due to its bioactive compounds, such as epicatechin and mitraphylline, similar to polyphenolic compounds like quercetin, which has demonstrated comparable effects on ROS reduction and UPR modulation in skeletal muscle cells [59]. These findings align with prior reports that UT extract protects cells from oxidative stress and apoptosis in various models [30,60].

It is known that an increase in ROS production in cells can lead to oxidative stress, which often results in improper protein folding in the endoplasmic reticulum (ER), potentially causing ER stress and the initiation of the unfolded protein response (UPR) [25,29,61]. Thus, it became interesting to assess whether incubating the cells with PA would be related to ER stress and whether treatment with UT aqueous extract could interfere with molecular events involved in this process.

Following the reduction in ROS, we investigated whether UT modulated UPR signaling. In order to induce ER stress in myoblast or myotube cultures, Tg was used as a positive control. This compound inhibits the activity of the endoplasmic reticulum calcium ATPase (SERCA ATPase) enzyme and promotes a reduction in calcium (Ca^2+^) ions in the ER lumen [29,41]. Additionally, Tn was applied, which interferes with the inhibition of glycosylation at the N-terminal of proteins, promoting misfolding [40,41].

Alternatively, the stress inhibitor TUDCA, a bile salt which modulates caspase expression and cell death, was used [62,63].

Thus, the effect of the UT aqueous extract on cells incubated with thapsigargin (Tg) and tunicamycin (Tn) was studied to investigate further its involvement in attenuating ER stress and to compare it with that of classic ER stress protectors, such as the bile salt TUDCA.

Also, considering that myotubes better represent the complexity of muscle tissue in vivo, it was decided to use only myotube cultures to study events involved in ER stress, as well as in the UPR pathway and insulin signaling, through the analysis of the expression of the genes ATF4, CHOP, and IRS-1, as well as the proteins encoded by these genes.

It was found that the incubation of cultures with PA increased the expression of ATF4 and CHOP. In this regard, it was observed that treatment with the UT aqueous extract reduced, on average, 3.5 times the expression of ATF4 in cultures incubated with (Tn), compared to cultures incubated only with (PA).

These transcriptional changes were corroborated at the protein level by Western blotting. No detectable expression of the protein was observed in cultures incubated with PA or Tg and treated with UT aqueous extract under the therapeutic condition. The same was observed for the evaluation of CHOP expression, where no protein expression was detected after therapeutic treatment in myotube cultures.

These results suggest that therapeutic treatment with UT may interfere in the pathways involving ATF4 and CHOP, whose mechanism is related to DNA damage repair and regulation of cell death through apoptosis [13,25].

Also, it was shown in this study that 20 µM Tg or Tn, as well as 500 µM PA, in addition to inducing ER stress and the UPR in cells, were also capable of decreasing IRS-1 expression by 3.5-fold in myotubes, affecting insulin substrate signaling at the IRS-1 receptor, which is considered the onset of insulin resistance [64,65].

On the other hand, UT aqueous extract treatment increased IRS-1 expression by around 3-fold in myotube cultures that received the therapeutic treatment for 6 h, after incubation with the PA, Tg, or Tn, suggesting a potential role of UT in improving markers associated with insulin signaling, under ER stress conditions. These results also align with in vivo studies, which demonstrate that restored IRS-1 expression improves insulin sensitivity in hepatocytes [31], confirming the therapeutic effect of UT in insulin resistance conditions.

This study presents some limitations that should be considered. The experiments were performed exclusively in an in vitro C2C12 myotube model, which does not fully reproduce the physiological complexity of insulin resistance and metabolic dysfunction in vivo. In addition, isolated secondary metabolites from the extract were not individually evaluated, which limits the direct identification of the compounds responsible for the observed biological effects. Future studies involving bioassay-guided fractionation, in vivo models and clinical investigations are necessary to further characterize the mechanisms and therapeutic relevance of *Uncaria tomentosa* in metabolic disorders.

Nevertheless, in summary, our findings provide compelling evidence that the UT aqueous extract modulates the UPR pathway decreasing the expression of ATF4 and CHOP, and restores IRS-1 levels in palmitate-induced myotubes.

The present findings demonstrate that the aqueous extract of *Uncaria tomentosa* exerts modulatory effects on markers associated with ER stress and insulin resistance in an in vitro skeletal muscle cell model. Although the results suggest potential biological relevance, additional in vivo and clinical studies are necessary to confirm the pharmacological applicability and therapeutic significance of these observations.

## 5. Conclusions

The results of this study confirm the antioxidant, anti-apoptotic and insulin-sensitizing effects of the UT extract by reducing ER stress markers (ATF4/CHOP) and restoring IRS-1 expression in differentiated myotubes. These findings support the therapeutic potential of UT as an adjuvant in metabolic conditions such as T2D, and lay the groundwork for future in vivo investigations and clinical exploration.

## Figures and Tables

**Table 1:** Classification of cytotoxicity related to the percentage of cell viability [37].

Cytotoxicity	Cell Viability (%)
Non-cytotoxic	≥90%
Slightly	Between 80 and 89%
Moderately	Between 50 and 79%
Severely	≤50%

**Table 2:** Oligonucleotide sequences. The expression of different genes encoding target proteins involved in the UPR pathway (ATF4 and CHOP), myogenesis (DES, Myf5, MyoD1, Myog, Myf6), insulin signaling pathway (IRS-1), and the expression of the constitutive gene (GAPDH) was assessed.

Targets	Sequences (5’–3’)		References
	Forward	Reverse	
ATF4	AACCTCATGGGTTCTCCAGCGA	CTCCAACATCCAATCTGTCCCG	[42,43]
CHOP	AAGCCTGGTATGAGGATCTGCAC	TTCCTGGGGATGAGATATAGGTG	
IRS-1	TGTCACCCAGTGGTAGTTGCTCC	CTCTCAACAGGAGGTTTGGCATG	[43,44]
PAX7	CTCCCTCTGAAGCGTAAGCA	GGGTAGTGGGTCCTCTCGAA	[45,46]
DES	GGAAGCCGAGGAATGGTACA	TCGATCTCGCAGGTGTAGGA	
Myf5	GGTGGAGAACTATTACAGCCTGC	ACAGTAGATGCTGTCAAAGCTGC	[47,48]
MyoD1	GCACTACAGTGGCGACTCAGAT	TAGTAGGCGGTGTCGTAGCCAT	
Myog	CCATCCAGTACATTGAGCGCCT	CTGTGGGAGTTGCATTCACTGG	
Myf6/MRF4	GCCAAGTGTTTCGGATCATTCCA	CTGTCCACGATGGAAGAAAGGC	
GAPDH	CATTTCCTGGTATGACAACGAC	GCACAGGGTACTTTATTGATGG	[49]

## Data Availability

Previously reported data were used to support this study and are available at DOI: 10.1186/s12906-023-04204-4. This prior study of our group is cited as the reference [29].

## Supplementary Materials

The following supporting information can be downloaded at: https://www.mdpi.com/article/10.3390/cimb48060624/s1.

## Abbreviations

AFU	Arbitrary fluorescent units
ANOVA	Analysis of variance
ATCC	American Type Culture Collection
ATF4	Activating Transcription Factor 4
BSA	Bovine serum albumin
cDNA	Complementary deoxyribonucleic acid
CHOP	C/EBP homologous protein
DCFDA	Dichlorodihydrofluorescein diacetate
Des	Desmin
DMEM	Dulbecco’s Modified Eagle Medium
DMSO	Dimethyl sulfoxide
dNTPs	Deoxynucleotide triphosphates
DTT	Dithiothreitol
eIF2a	Eukaryotic translation initiation factor 2 alpha
ER	Endoplasmic reticulum
GAPDH	Glyceraldehyde-3-phosphate dehydrogenase
IRS-1	Insulin receptor substrate 1
JNK	Jun N-terminal kinase
MRF4	Myogenic Regulatory Factor 4
mRNA	Messenger ribonucleic acid
MTT	3-(4,5-dimethylthiazol-2-yl)-2,5-diphenyltetrazolium bromide
Myf5	Myogenic factor 5
Myf6	Myogenic factor 6
MyoD1	Myogenic differentiation 1
Myog	Myogenin
PA	Palmitate
PAX7	Paired box 7
PBS	Phosphate buffer saline
PCR	Polymerase chain reaction
PERK	Protein kinase R-like ER kinase
PI3K	Phosphoinositide 3-kinase
RNA	Ribonucleic acid
ROS	Reactive oxygen species
SERCA	Sarcoendoplasmic reticulum calcium ATPase
T2D	Type 2 diabetes
Tg	Thapsigargin
Tn	Tunicamycin
TNF-α	Tumor Necrosis Factor alpha
TUDCA	Tauroursodeoxycholic acid
UT	*Uncaria tomentosa*
