# Supplementary material for: Modulatory Activity of Uncaria tomentosa Extract in the Expression of Proteins Involved in the Unfolded Protein Response and Insulin Resistance
Source: Curr Issues Mol Biol. 2026 Jun 16;48(6):624. doi: 10.3390/cimb48060624 (PMC13298498; doi:10.3390/cimb48060624)
Supplement: Supplementary file 1 [file cimb-48-00624-s001.zip › cimb-4321908-supplementary.pdf]

Article

Modulatory activity of *Uncaria tomentosa* extract in the expression of proteins involved in the unfolded protein response and insulin resistance

Bruna Freitas Marchi [1,2,*], Vittoria de Lima Camandona [3], Athirson Moraes Chanavat [2], Gustavo Roncoli Reigado [2], Carla Roberta de Oliveira Carvalho [4], Felipe Santiago Chambergo [2] and Viviane Abreu Nunes [2,*]

[1] Department of Chemistry and Molecular Biology, University of Gothenburg (GU), 413 90 Göteborg, Sweden
[2] Laboratory of Skin Physiology and Tissue Bioengineering, School of Arts, Sciences and Humanities, University of Sao Paulo (EACH-USP), São Paulo 03828-000, Brazil
[3] Laboratory of Aging and Molecular Biology, School of Arts, Sciences and Humanities, University of Sao Paulo (EACH-USP), São Paulo 03828-000, Brazil
[4] Department of Physiology and Biophysics, Institute of Biomedical Sciences, University of São Paulo (ICB-USP), São Paulo 05508-000, Brazil
* Correspondence: bruna.marchi@gu.se (B.F.M.); vanunes@ib.usp.br (V.A.N.)

SUPPLEMENTARY MATERIAL

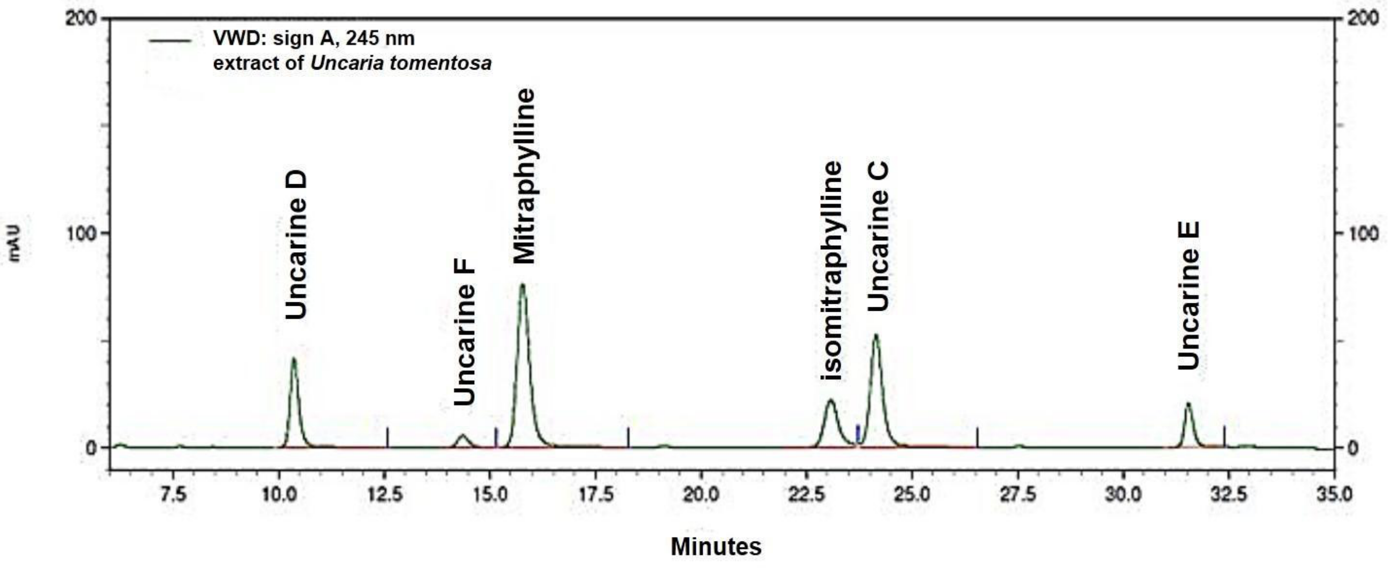

Figure S1: HPLC chromatogram of the *Uncaria tomentosa* crude extract used in the experiments. The analysis identified some oxindole alkaloids, including uncarine D, uncarine F, isomitraphylline, uncarine C, uncarine E and mitraphylline, the latter corresponding to 5.97% of the extract and representing one of the major oxindole alkaloids. The chromatographic profile was monitored at 245 nm over 35 min, with signal intensity expressed in milli-Absorbance Units (mAU). Adapted from Freitas-Marchi *et al*., 2023.

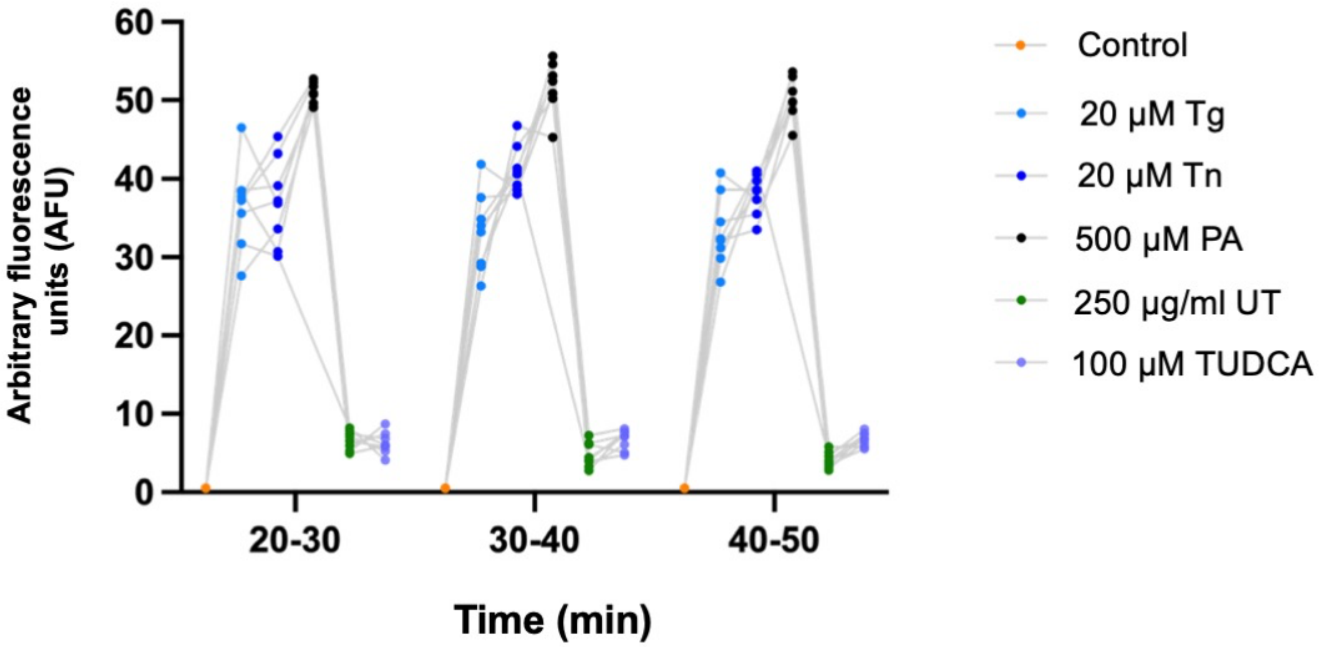

Figure S2. Kinetics of DCFDA fluorescent probe in myoblast cultures. Evaluation of the reaction time of the ER stressors thapsigargin (Tg), tunicamycin (Tn) and palmitate (PA) incubated for 24 h, or the treatment of the non-stressors *Uncaria tomentosa* (UT) and tauroursodeoxycholic acid (TUDCA) incubated for 6 h.
